# Reliability and validity of the center for epidemiologic studies-depression scale in screening for depression among HIV-infected and -uninfected pregnant women attending antenatal services in northern Uganda: a cross-sectional study

**DOI:** 10.1186/s12888-014-0303-y

**Published:** 2014-11-22

**Authors:** Barnabas K Natamba, Jane Achan, Angela Arbach, Thomas O Oyok, Shibani Ghosh, Saurabh Mehta, Rebecca J Stoltzfus, Jeffrey K Griffiths, Sera L Young

**Affiliations:** Division of Nutritional Sciences, Cornell University, Ithaca, NY 14853 USA; Department of Public Health, Faculty of Medicine, Gulu University, Gulu, Uganda; PRENAPS Uganda, Kampala, Uganda; Department of Pediatrics and Child Health, Makerere University, Kampala, Uganda; Weill Cornell Medical College, New York, NY 10021 USA; Department of Mental Health, Faculty of Medicine, Gulu University, Gulu, Uganda; Friedman School of Nutrition Science and Policy, Tufts University, Boston, MA 02155 USA; Department of Public Health and Community Medicine, School of Medicine, Tufts University, Boston, MA 02155 USA

**Keywords:** Pregnancy, Major depression, Prenatal depression, Uganda, Africa, Screening

## Abstract

**Background:**

There are limited data on the prevalence and approaches to screening for depression among pregnant women living in resource poor settings with high HIV burden.

**Methods:**

We studied the reliability and accuracy of the Center for Epidemiologic Studies Depression (CES-D) scale in 123 (36 HIV-infected and 87 -uninfected) pregnant women receiving antenatal care at Gulu Regional Referral Hospital, Uganda. CES-D scores were compared to results from the psychiatrist-administered Mini-International Neuropsychiatric Interview (MINI) for current major depressive disorder (MDD), a “gold standard” for assessing depression. We employed measures of internal consistency (Cronbach’s alpha), and criterion validity [Area Under the Receiver Operating Characteristic Curve (AUROC), sensitivity (Se), specificity (Sp), and positive predictive value (PPV)] to evaluate the reliability and validity of the CES-D scale.

**Results:**

35.8% of respondents were currently experiencing an MDD, as defined from outputs of the MINI-depression module. The CES-D had high internal consistency (Cronbach’s alpha = 0.92) and good discriminatory ability in detecting MINI-defined current MDDs (AUROC = 0.82). The optimum CES-D cutoff score for the identification of probable MDD was between 16 and 17. A CES-D cutoff score of 17, corresponding to Se, Sp, and PPV values of 72.7%, 78.5%, and 76.5%, is proposed for adoption in this population and performs well for HIV-infected and -uninfected women. After adjusting for baseline differences between the HIV subgroups (maternal age and marital status), HIV-infected pregnant women scored 6.2 points higher on the CES-D than HIV-uninfected women (p = 0.032).

**Conclusions:**

The CES-D is a suitable instrument for screening for probable major depression among pregnant women of mixed HIV status attending antenatal services in northern Uganda.

**Electronic supplementary material:**

The online version of this article (doi:10.1186/s12888-014-0303-y) contains supplementary material, which is available to authorized users.

## Background

Since the first Global Burden of Disease (GBD) report was released in 1996 [1], the impacts that HIV infection and major depressive disorders (MDDs) have had on medical and public health systems have greatly increased. The 2010 GBD report indicated that HIV infection has risen from being the 33rd to the 5th contributor to the global burden of disease, while MDDs have risen from being the 15th leading cause to being the 11th [2]. While there has been a surge of information and strategies for managing or preventing HIV infection, practical strategies for managing MDDs remain limited, particularly in resource poor settings.

In Sub-Saharan Africa (SSA), the reported prevalence of MDDs among adult people living with HIV (PLHIV) ranges broadly, from 71.3% in Zambia [3] to 47% in Uganda [4], 43.7% in South Africa [5], and 30% in Zimbabwe [6]. A number of biological and psychosocial factors and psychiatric co-morbidities help explain why persons with HIV are at a higher risk of depression [7]. Moreover, depression has been associated with increased rates of HIV disease progression and mortality [8], inequity in decision-making and relationship power among heterosexual couples [9], and AIDS-related stigma [10].

Reports indicate that as many as 30% of PLHIV develop MDDs during the course of their illness [11,12]. The majority of studies reporting the prevalence or incidence of depressive symptoms among PLHIV in SSA have not compared rates among PLHIV to those among HIV-negative persons. Both depression [13-15] and HIV [7,16] disproportionately affect more women than men. Limited data from SSA settings [17] and recent data from the US [18] have examined the cross-sectional linkage between maternal HIV infection and depression prevalence during pregnancy. No study has examined the differences in the trajectory of depressive symptoms among HIV-infected and -uninfected pregnant women. In the sole cross-sectional study that compared the prevalence of depressive symptoms among newly tested HIV-positive and HIV-negative pregnant women in South Africa, no association between HIV infection status and depressive symptoms severity was found [17]. A similar result of no difference in depressive symptoms by HIV serostatus was reported for US women attending an obstetrics/gynecology clinic in urban Philadelphia [18]. It is, therefore, unclear whether the prevalence or incidence of major depression or depressive symptoms differs between HIV-infected and -uninfected pregnant women in SSA. Integrating screening, referral, and treatment for major depression into programs targeting HIV-infected and -uninfected pregnant women in SSA could help fill this gap.

Screening for depression among pregnant women is of specific interest, because this would facilitate early detection of depressive symptoms and the referral and treatment of women with probable major depression. Depression may affect pregnant women’s functionality, capacity to care for themselves, and ability to utilize household and community level resources. Depressed women may not be able to (1) acquire and prepare food, (2) attend antenatal care (ANC) services, and, for those that are HIV positive, (3) utilize services aimed at preventing mother to child transmission of HIV (PMTCT). Depression during pregnancy is a risk factor for postnatal depression [19] and may have direct [20] or indirect (through its influence on postnatal depression; [21]) impacts on mother-child interactions and the infants’ emotional and behavioral development. It is important that pregnant women with or at risk of depression are screened and appropriately treated before it becomes too late to intervene. Antenatal care (ANC) clinics in regional, district, and lower level health centers in resource poor settings, such as northern Uganda, provide potential entry points for screening, referral and treatment of pregnant women with or at risk of depression.

In order to appropriately identify pregnant women with or at risk of depression, there is a need for a reliable and valid screening tool. A recent review of depression screening tools used in low and middle-income countries did not recommend any specific instrument [22]. The Center for Epidemiologic Studies-Depression (CES-D) [23] is an attractive screening tool for depression because it covers all of the common symptoms of major depression, i.e. depressive mood, feelings of guilt and worthlessness, psychomotor retardation, loss of appetite, and sleep disturbance. The CES-D consists of 20 items that are designed to measure self-reported depressive symptoms in the prior two weeks. Each of the 20 items is assigned a value of 0–3, with four items positively worded and reverse scored. The total score is computed by summing the 20 items, such that the range of scores is 0 to 60. A score of ≥16 in the general US population is used to suggest the presence of probable MDD. Elsewhere, the cutoff will vary based on the prevalence of each of the 20 items in the population of interest, the method of the scale’s administration (whether as a self report or interviewer administered), and the extent of adaptation (e.g. translation into another language before administration) that goes into making the CES-D contextually relevant. Hence, it is important that the scale be validated before it is used in new populations.

Within SSA the CES-D has been validated among adult populations in Zambia [3] and South Africa [5] by comparing results of the CES-D to those obtained using a ‘gold standard’ for depression assessment, the depression module of the Mini-International Neuropsychiatric Interview (MINI-D). The MINI (and the MINI-D component as well) is a short, structured diagnostic interview that was developed in 1990 by psychiatrists and clinicians in the United States and Europe for DSM-IV psychiatric disorders [24].

Although the CES-D has been used among PLHIV in Uganda [4,25] we did not find studies documenting the scales validity in this context. Furthermore, these two studies were conducted among HIV-infected adults only, making it difficult to compare findings from such populations to those of HIV-uninfected populations. Thus, the aim of this study was to assess the reliability and validity of the CES-D as a relatively simple screening tool for probable major depression among HIV-infected and -uninfected pregnant women in northern Uganda.

## Methods

### Design, setting and participants

Data were collected from the 10th of October 2012 to the 21^st^ of December 2012, within the context of the Prenatal Nutrition and Psychosocial Health Outcomes (PreNAPs) study. The PreNAPs study is a longitudinal observational study that seeks to describe the prevalence and trajectory of depressive symptoms among HIV-infected and -uninfected pregnant women attending ANC services in northern Uganda.

HIV-infected and -uninfected participants were consecutively and separately recruited from the ANC clinic of Gulu Regional Referral Hospital (GRRH) in Gulu, northern Uganda, a busy primary care clinic that receives more than 400 initial antenatal visits monthly. As at other public medical facilities in Uganda, all services at GRRH, including medications, care for pregnant women with HIV, etc., are offered free of charge. Consistent with Ugandan national policy, all HIV-infected women received prophylactic ARVs.

HIV-infected and -uninfected pregnant women who presented at ANC between 10 and 26 weeks of gestation and resided within 30 km of GRRH were invited to participate. HIV infected women were oversampled to achieve a minimum ratio of 1 HIV infected: 2 HIV uninfected participants, thus our sample has a higher proportion of HIV infected women than the 10.3% age adjusted prevalence of HIV previously observed at ANC clinics in northern Uganda [26].

### Data collection

All study instruments were translated by local research staff into Acholi and Langi, the two predominant and closely related Luo languages that are spoken in the study communities. The questionnaires were then back-translated into English by the same team, and discrepancies in conceptual and semantic equivalence were resolved through discussion involving all the translators, the research assistants, and the GRRH psychiatrist (TOO).

Maternal HIV status was determined at the ANC clinic based on the Ugandan government’s HIV counseling and testing guidelines [27] and prior to enrollment into this study. Gestational age at recruitment was based on first day of the last month of menstrual period. Wealth was operationalized based on the pregnant women’s report of their total household income divided by the number of household members.

The CES-D was administered by members of the PreNAPs study team (either a nutritionist, midwife, or general nurse) after baseline health and demographics data had been collected. Then, on the same day, respondents underwent a diagnostic interview by the GRRH psychiatrist (TOO), who was blinded to the results of the CES-D screening interview, using the MINI-D.

One hundred and thirty-five consecutive pregnant women were approached to participate in the MDD sub-study of PreNAPs, and 125 (92.6%) consented to participate. Ten participants did not participate in the study upon request due to “lack of time.” Of the 125 participants who agreed to take part in the study, two had incomplete survey data and were excluded from the final analysis; the rest (n = 123, 36 HIV-infected and 87 HIV-uninfected) participated in both the CES-D and MINI-D interviews.

### Analysis

Data were analyzed using STATA version 12 (StataCorp LP, College Station, TX). We compared HIV-infected and -uninfected pregnant women with regard to measured baseline demographic and socioeconomic variables. To test for baseline differences between HIV -infected and -uninfected pregnant women, we used Pearson’s chi-squared test for categorical variables, Student’s t-tests, and the nonparametric equality-of-medians test for continuous variables. Study participants were categorized into cases and non-cases of currently experiencing a MDD based on the MINI-D module outputs. Median total scores on the CES-D were then compared against the MINI-D diagnoses of current MDD using the Wilcoxon rank-sum test. We fit a linear regression model, adjusting for baseline correlates of maternal HIV infection status, to determine differences in baseline CES-D scores between HIV-infected and -uninfected pregnant women.

We assessed the reliability of the CES-D scale using the Cronbach’s test for internal consistency. Cronbach’s alpha coefficients of 0.7 or greater were considered to be reliable [28]. We determined the criterion validity of the CES-D, i.e. the extent to which measurements obtained using the CES-D concurred with those determined with an established diagnostic test or gold standard [29], by comparing scores on the CES-D with psychiatrist administered MINI-D outputs. Further, we used a non-parametric method, Area Under the Receiver Operating Characteristic Curve (AUROC) analysis, to test the CES-D’s criterion validity. We selected the optimum CES-D cutoff scores that concurrently maximized sensitivity (Se), specificity (Sp) and positive predictive values (PPV) when compared to the psychiatrist administered MINI-D assessment.

## Results

### Characteristics of the study participants

Characteristics of the studied sample are presented in Table 1. HIV-infected and -uninfected women did not differ in terms of their gestational age at assessment, the predominant language they spoke, level of education, occupation, or monthly household income per capita. HIV-infected participants were 3.2 years older (p = 0.001) and 21.9% less likely to be in a married or cohabiting relationship (p = 0.002) than HIV-uninfected participants. Based on the results of the MINI-D interview, a greater proportion of HIV-infected participants (52.9%) were more likely to be classified as having an MDD than HIV-uninfected participants (28.7%; p = 0.011). After adjusting for maternal age and marital status, HIV-infected pregnant women scored 6.2 points higher on the CES-D than HIV-uninfected participants (p = 0.032)

**Table 1:** **Characteristics pregnant women participating in the study to validate the Center for Epidemiologic Studies-Depression (CES-D) scale in northern Uganda, October 2012–December 2012**

**Variable**	**Estimate**	**Overall (n = 123)**	**HIV- (n = 87)**	**HIV + (n = 36)**	**HIV- vs. HIV + p value**
Gestational age at interview, weeks	Mean (s.e.)	18.3 (0.36)	18.3 (0.43)	18.5 (0.66)	n.s.***
Participant speaks Acholi or Langi	%	91.9%	91.9%	91.7%	n.s.
Participants’ age, years	Mean (s.e.)	24.3 (0.44)	23.3 (0.49)	26.5 (0.76)	0.001
Woman identifies as married or cohabiting	%	87.0%	93.1%	71.2%	0.002
Primary or no education	%	53.7%	49.4%	63.9%	n.s.
Woman identifies as housewife	%	50.4%	51.7%	47.2%	n.s.
Monthly household income per capita (in Uganda Shillings)*	Mean (s.e.)	87,005 (9,229)	93,105 (10,991)	72,433 (16,988)	n.s.
MINI-defined current major depressive disorder	%	35.8%	28.7%	52.9%	0.011
Unadjusted CES-D score	Mean (s.e.)	17.1 (1.24)	14.6 (1.42)	23.2 (2.21)	0.001
Adjusted CES-D score**	Mean (s.e.)	n/a	15.3 (1.44)	21.5 (2.33)	0.032

*At the time the study was done, 1 US Dollar was equivalent to 2,500 Ugandan Shillings.

**Differences in CES-D scores between HIV-infected and -uninfected pregnant women after adjusting for age and marital status.

***n.s. refers to non-significant.

.

### Reliability and validity of the CES-D scale

**Figure 1:**
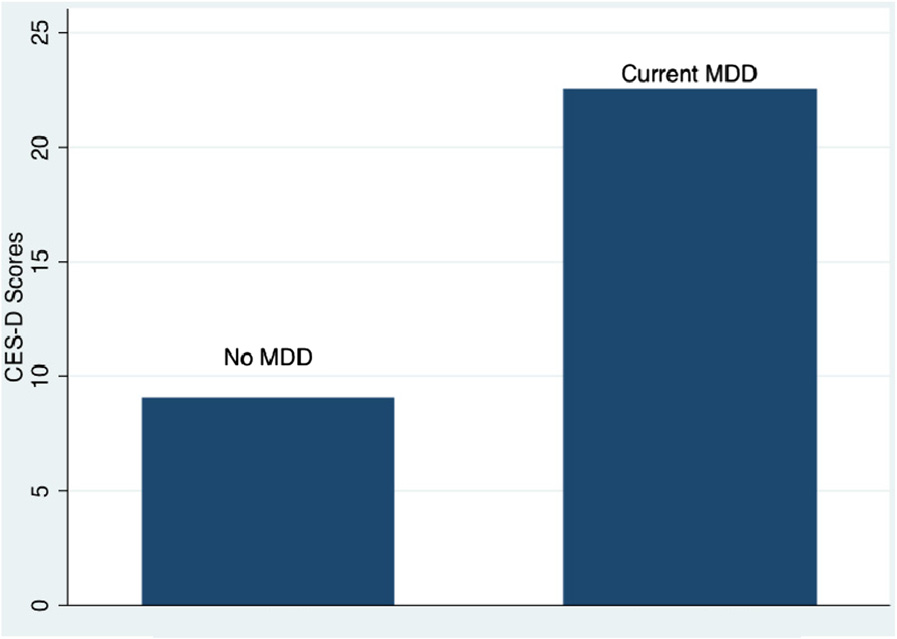
**Median CES-D scores by psychiatrist diagnosed current MDD among HIV-infected and -uninfected pregnant women (n 123) attending antenatal care clinics in northern Uganda, October 2012–December 2012.**

**Figure 2:**
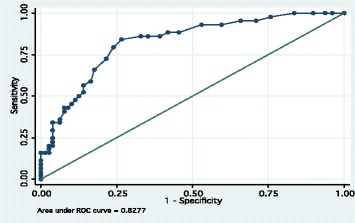
**Area under the receiver operating characteristic curve (AUROC) for CES-D scores for diagnosis of current MDD among HIV-infected and -uninfected pregnant women (n 123) attending antenatal care clinics in northern Uganda, October 2012–December 2012.**

The internal consistency of the CES-D was high (Cronbach’s alpha = 0.92). As expected, CES-D scores were higher among MINI-defined current MDD cases than non-MDD cases (Figure 1, P <0.001). Furthermore, the CES-D discriminated well between current MDD cases and non-cases (Figure 2, AUROC = 0.82).

**Figure 3:**
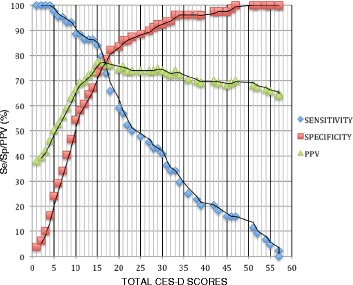
**Sensitivity, specificity, and positive predictive values for the CES-D by cutoff scores, for diagnosis of current MDD among HIV-infected and -uninfected pregnant women (n 123) attending antenatal care clinics in northern Uganda, October 2012–December 2012.**

Se, Sp, and PPV at each CES-D score indicated that the optimum CES-D cutoff score for current MDD was somewhere between 16 and 17 (Additional file 1: Table S1 & Figure 3). With a cutoff of 16, the corresponding Se, Sp and PPV values of 79.6%, 75.9%, and 77.2% were obtained. When a cutoff of 17 was selected, Sp increased by 2.6% to 78.5%, PPV dropped slightly by 0.7% to 76.5%, and Se drops by 7.1% to 72.7%.

**Figure 4:**
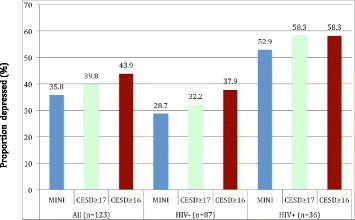
**Proportion of the sample classified as probable MDD cases at CES-D cutoff point of ≥16 or ≥17 in comparison to results from the psychiatrist diagnosed current MDD among HIV-infected and -uninfected pregnant women (n 123) attending antenatal care clinics in northern Uganda, October 2012–December 2012.**

Among HIV-infected participants, selecting a CES-D score cutoff value of either 16 or 17 did not affect the estimated prevalence of probable major depression (58.3%; Figure 4). On the other hand, selecting a cutoff value of 16 or 17 affected the estimated prevalence of depression among HIV-uninfected women. At the CES-D cutoff value of 16, the estimated prevalence of probable major depression was 9.2% higher than results obtained with the MINI-D (37.9% vs. 28.7%; Figure 4). A cutoff value of 17, however, provided a result that was much closer to results from the psychiatric assessment (32.2% vs. 28.7%; Figure 4).

## Discussion

Overall, the interviewer-administered CES-D scale, as validated in this study, was a reliable and valid instrument for screening for prenatal depression among pregnant women attending ANC services in northern Uganda.

The high Cronbach’s alpha (0.92) indicated that subjects in this study were consistent in their responses. This Cronbach value is similar to that reported by the original author of the self-reported CES-D [23] and is slightly higher than those reported among other studies documenting the reliability of interviewer-administered CES-D scales in other African settings [3,5]. For instance, among patients with tuberculosis (TB) and HIV in primary care in Zambia, the Cronbach alpha for an interviewer-administered CES-D was 0.84 [3].

The AUROC, a measure of the accuracy of discriminating between two groups, was 0.82, allowing us to use the CES-D to differentiate women with current MDDs from those without this condition (Figure 2). Our result is similar to AUROC values reported from CES-D validation studies conducted among HIV infected persons in South Africa (AUROC = 0.76; [5]) and TB and HIV patients in Zambia (AUROC = 0.78; [3]).

A highly sensitive test is needed for screening examinations in routine clinical care to identify potential cases, while a highly specific test is preferred for confirmatory purposes [30]. Good screening tests are expected to minimize the number and proportion of false positive results and, as such, have a high PPV. A test with high PPV is in part valued, because it reduces the expense and risks related to further evaluation of false positive tests, especially in settings with limited resources [30]. We employed a combination of Se, Sp, and PPV to arrive at the optimum CES-D cutoff score for probable major depression in our sample, a value that was between 16 and 17 (Figure 3).

A cutoff score of 16 had a relatively high Se and slightly higher PPV values than a cutoff score of 17 (Se 79.6% vs. 72.7%, PPV 77.2% vs. 76.5%; Additional file 1: Table S1). Using a cutoff of 16 in future studies, therefore, may help maximize the rate of truly positive results, permitting appropriate classification of a higher proportion of persons that actually have depression. On the other hand, a cutoff score of 16 had a lower Sp than a cutoff score of 17 (Sp 75.9 vs. 78.5; Additional file 1: Table S1).

As noted above, a CES-D cutoff score of 17 provided a higher Sp than the cutoff of 16 and, thus, greater ability to minimize the proportion of false positive cases of probable major depression. This cutoff also allowed us to attain prevalence rates that were closer to those identified through a psychiatrist-administered MINI-D interview. The CES-D cutoff of 17 resulted in 39.8% of the women to be classified as having a probable MDD, versus 43.9% using the cutoff score of 16. The percentage of women with probable depression at the 17 cutoff value (39.8%) was much closer to the prevalence of current MDD (35.8%) obtained with the MINI-D assessment (Figure 4). However, as shown in Figure 4, selection of a cutoff score of 17 appeared most helpful when screening HIV-negative respondents. Among HIV-positive respondents, the same percentage of respondents with a probable MDD (58.3%) was reached irrespective of whether a cutoff of 16 or 17 was used. Our information, therefore, suggests that using a cutoff of 17 is appropriate for both HIV-infected and -uninfected pregnant women, and we plan to adopt this cutoff for our ongoing study activities and reporting of the prevalence and incidence of probable major depression in the PreNAPs cohort.

The cutoff score of 17 for this interviewer administered CES-D scale is comparable to the cutoff score of 16 for the self-reported CES-D suggested by Radloff for the general US population [23]. In contrast, our cutoff value is much lower than scores obtained with the interviewer-administered CES-D study among HIV and TB patients in Zambia. In the Zambia study, a cutoff value of 22 was indicated to have a Se of 73% and PPV value of 76% when compared to MINI-D outputs [3]. The higher cutoff value obtained in that study may relate to the fact that their population, unlike ours that was predominantly HIV-uninfected, was only composed of HIV and TB infected participants. Furthermore, the MINI-D module for our study was administered by a medical psychiatrist (TOO), and mental health clinical assistants administered the MINI-D in the Zambia study.

Our data do not support the findings of others in South Africa [17] and the US [18], where depressive symptoms do not differ by pregnant women’s HIV status. After adjusting for maternal age and marital status, HIV-infected pregnant women in our study scored 6.2 points higher on the CES-D than HIV uninfected participants (Table 1, p = 0.032). However, the focus of this study was on validation of the CES-D instrument, and we caution against over-interpretation of this finding. We note that the HIV-infected women may have had adverse life experiences, such as the loss of their partners or family members due to HIV, or may have suffered from stigma, and these factors may be more powerful proximal determinants of differences in CES-D scores than the HIV infection itself. Given the number of HIV-infected women (n = 36) compared to HIV-uninfected participants (n = 87) in this study, we did not have sufficient power to fully test the hypothesis that HIV-infected women scored higher on the CES-D than -uninfected participants. In the future, larger studies will need to examine the hypothesis that CES-D scores and the prevalence and incidence rate of depression differ by pregnant women’s HIV-status. Such studies will also need to adjust for other correlates of depression, in addition to maternal age and marital status, such as food insecurity and women’s access to different forms of social support [31].

Our study has some limitations. For one, the generalizability of our findings may be limited. As noted above, it is possible that the CES-D performs differently among HIV-infected and -uninfected pregnant women. Our data, however, do suggest that an identical cutoff value of 17 is appropriate for the CES-D screening tool in both HIV-infected and -uninfected populations. We also note that the CES-D was administered to women at about 18 weeks of gestation (Table 1), and we cannot comment on how the cutoff scoring might have changed through the entire course of pregnancy. In addition, although the MINI-D module has been used successfully among different subpopulations in Uganda [32-34], we found no studies that, as recommended by its inventors [24], aimed to validate or culturally adapt it in ways that go beyond language translation. Furthermore, this study was done at a higher-level health facility, and pregnant women attending GRRH may differ from the general northern Uganda population (in terms of education, rural vs. urban residence, access to information, and other factors), such that our findings may not be widely generalizable.

In spite of these limitations, these data suggest that the CES-D as used in the PreNAPs study is a reliable and valid measure of depressive symptoms in this population. To expand the applicability of the CES-D within this context, future studies could assess the reliability and validity of the CES-D in the general northern Uganda population. We believe there would be merit in using a relatively large sample size that can permit separate assessment of the CES-D screening tool’s reliability and validity among HIV-infected and -uninfected pregnant women.

In the short term, interventions to mitigate prenatal depression in this vulnerable population are needed, given the high prevalence of psychiatrist diagnosed major depression (28.7% among HIV-uninfected, vs. 52.9% in HIV-infected subgroups) and the likely adverse impacts of depression on women [19] and their infants [20,21]. Modifications to current mental health screening policies, especially among PMTCT attendees, may help with identification and treatment of depressed pregnant women. Most Ugandan regional referral hospitals have mental health departments. However, opportunities for screening, referral and follow-up of affected patients are usually missed due to limited staffing, remuneration, motivation, and retention of skilled health workers. Current national ANC guidelines are silent about integrating screening for mental health problems such as depression. The high prevalence of MDD that we observed in this study suggests the need for revision of existing Ugandan ANC guidelines to incorporate screening, treatment and referral of pregnant women for depression. The World Health Organisation (WHO) recommends integrating mental health activities into ANC programs [35], and approaches for doing this in resource-poor settings have been identified, for example through the use of WHO mental health Gap Action Program guidelines [36]. The CES-D is a brief, valid, and simple screening tool that could be used to identify pregnant women with or at risk of depression.

## Conclusions

The CES-D scale, administered by non-psychiatrists, was found to be a reliable and valid screening tool in a population of women of mixed HIV status receiving ANC, when compared to a psychiatrist-administered and structured MINI-D interview. Given the high prevalence (35.8%) of psychiatrist-diagnosed depression in this population, there is need to adopt a reliable and valid screening tool for depression (such as the CES-D), further evaluation, and the treatment of pregnant women with elevated depressive symptoms. More research will be needed, however, to assess the time demands and overall programmatic experience of integrating care for depression into current ANC service delivery platforms.

### Ethical considerations

The study protocol was approved by Cornell University Institutional Review Board and Gulu University Institutional Review Committee. Permission to conduct the study in Uganda was obtained from the Ugandan National Council for Science and Technology. Written informed consent was obtained from all study subjects before enrollment. Patients identified to be severely depressed, per the MDD module of the MINI, were referred for further assessment and treatment at GRRH.

## Additional file

Additional file 1: Table S1. Sensitivity, specificity, and positive predictive values for probable major depression at various CES-D cutoff scores of HIV-infected and -uninfected pregnant women (n 123) attending antenatal care clinics in northern Uganda, October 2012–December 2012.

## Abbreviations

ANC: Antenatal care
AUROC: Area under the receiver operating characteristic curve
CES-D: Center for epidemiologic studies- depression scale
GBD: Global burden of disease
GRRH: Gulu Regional Referral Hospital
MDD: Major depressive disorder
MINI-D: Mini-international neuropsychiatric interview- depression module
PMTCT: Prevention of mother to child transmission of HIV
PPV: Positive predictive value(s)
PreNAPs: Prenatal nutrition and psychosocial health outcomes study
Se: Sensitivity
Sp: Specificity
SSA: Sub-Saharan Africa
WHO: World Health Organisation
